# Quantitative assessment of ophthalmic viscosurgical device retention during phacoemulsification and aspiration: an ex vivo analysis

**DOI:** 10.1007/s00417-025-07073-4

**Published:** 2025-12-18

**Authors:** Ippei Watanabe, Hirotaka Hoshi, Kanna Cho, Hirokazu Mukuno

**Affiliations:** 1 Medical Affairs, Seikagaku Corporation, 1-6-1 Marunouchi, Chiyoda-ku, Tokyo, 100-0005 Japan; 2 Central Research Laboratories, Seikagaku Corporation, Higashiyamato, Tokyo Japan; 3 Clinical Development, Seikagaku Corporation, Chiyoda-ku, Tokyo, Japan; 4 Shinnagata Eye Hospital, Kobe, Hyogo Japan

**Keywords:** Chondroitin sulfate, Ophthalmic viscosurgical devices, Phacoemulsification, Residual volume, Soft shell technique

## Abstract

**Purpose:**

To quantify the amount of residual ophthalmic viscosurgicaldevice (OVD) during phacoemulsification and aspiration (PEA).

**Methods:**

A fluorescein-stained dispersive OVD consisting of 3% hyaluronic acid (HA) and 4% chondroitin sulfate (CS) was injected into porcine eyes in volumes of 0.05, 0.1, and 0.15 mL. Subsequently, a cohesive OVD containing 1% HA was injected, and the soft shell technique (SST) was used. Porcine eyes filled with 0.4 mL of dispersive OVD alone were also evaluated. PEA was performed for 0.5, 1, 2, 3, 4, and 5 min, and the amount of dispersive OVD remaining in the eye at each time point was quantified by measuring sulfate ions contained in the CS molecules.

**Results:**

Using the SST with 0.1 mL of dispersive OVD, the corneal endothelium was covered for up to 2 min of PEA, and approximately 60 μL of dispersive OVD remained. With the SST using 0.15 mL of dispersive OVD, approximately 60 μL of dispersive OVD remained 5 min after PEA, but using0.05 mL, less than 30 μL remained 0.5 min after PEA, indicating insufficient protection of the corneal endothelium. With 0.4 mL of the single agent, 60 μL of dispersive OVD remained in the eye, even after 5 min of PEA.

**Conclusion:**

The relationship between the injectate volume of dispersive OVD and the residual volume in the eye over the PEA time was demonstrated quantitatively. To perform SST properly, it is necessary to consider the amount of the dispersive OVD injected.

**Supplementary Information:**

The online version contains supplementary material available at 10.1007/s00417-025-07073-4.

## Abstract (highlights)

**Key messages:**

*****What is known***:**

- In most previous ex vivo studies, the time taken to remove dispersive ophthalmic viscosurgical devices (OVDs) from the eye was assessed qualitatively by visual inspection of fluorescein-stained OVDs.
- There has been no evaluation to date of the amount of residual OVD remaining in the eye during phacoemulsification and aspiration (PEA), relative to the injection volume of dispersive OVD using the soft shell technique.

*****What is new***:**

- By measuring sulfate ions in the chondroitin sulfate molecules contained in the dispersive OVD, we evaluated the amount of dispersive OVD in the eye during PEA.
- The relationship between the volume of dispersive OVD injected using the soft shell technique and the remaining volume of dispersive OVD in the eye at each PEA time can provide important information related to cataract surgery.

**Supplementary Information:**

The online version contains supplementary material available at 10.1007/s00417-025-07073-4.

## Introduction

Ophthalmic viscosurgical devices (OVDs) protect the corneal endothelium from contact with instruments or lens fragments and air bubbles generated during phacoemulsification and aspiration (PEA) in cataract surgery. In modern cataract surgery, OVDs are an essential tool for protecting the corneal endothelium, which does not regenerate once damaged. OVDs are primarily classified into two types: cohesive and dispersive [1–3]. Cohesive OVDs containing high-molecular-weight hyaluronic acid (HA) have high viscoelasticity and are useful for maintaining the anterior chamber space [4, 5]. Conversely, dispersive OVD, consisting of 3% HA and 4% chondroitin sulfate (CS), is retained in the anterior chamber during surgery because of its adhesive nature in comparison with cohesive OVDs [6, 7]. Therefore, the dispersive OVD can cover and protect the corneal endothelium from mechanical or heat injuries and free radical formation due to ultrasound during PEA [8].

These two types of OVDs have different physical properties and therefore behave differently in the eye during PEA. The soft shell technique (SST), reported in 1999, is a surgical procedure that utilizes the advantages of both cohesive OVD and dispersive OVD [9]. In SST, a dispersive OVD is first injected into the center of the anterior chamber, subsequently, a cohesive OVD is injected under the dispersive OVD. This causes the dispersive OVD to be pushed forward onto the corneal endothelium, forming a protective layer, while the cohesive OVD creates space in the anterior chamber. Computational fluid dynamics have also been used to demonstrate the intraocular behavior of two OVDs using the SST [10].

Premature removal of OVDs carries the risk of damaging endothelial cells. Therefore, it is necessary to be aware how much OVD is retained in the eye and for how long, during surgery. Although several studies have evaluated the intraocular retention of OVDs during surgery, it is difficult to accurately assess the amount of colorless, transparent OVD remaining in the eye in clinical practice. Furthermore, although some studies have evaluated the PEA time required for OVD removal in vivo and ex vivo using stained OVDs, the amount of OVD remaining has not been quantified. Previous studies have had issues with determining the time required to remove an OVD from the eye by visual observation, which can be subjective. Furthermore, since the injection volume and test conditions of OVDs vary from one publication to another, it is difficult to compare the residence time of OVDs in the eye, between publications. To address these issues, this study measured the quantity of CS in the HA/CS combination, to evaluate the remaining amount of dispersive OVD in porcine eyes during PEA.

## Materials and methods

### Materials

Shellgan^®^ (Santen Pharmaceutical Co., Ltd., Osaka, Japan) containing 3% HA and 4% CS was used as the dispersive OVD, and Sodium Hyaluronate Ophthalmic Viscoelastic Preparation 1% “SEIKAGAKU”^®^ (Santen Pharmaceutical Co., Ltd) containing 1%, high-molecular-weight HA was used as the cohesive OVD. Porcine eyes were purchased from Tokyo Shibaurazoki Corp. (Tokyo, Japan). The eyes were harvested from 6-month-old female or castrated pigs that are a hybrid of four swine breeds, Landrace, Yorkshire, Berkshire, and Duroc. In a previous study, the mean area of porcine corneas of ​​the same species as in this study was 136 mm^2^ [11].

### Removability of OVDs from the porcine anterior chamber during PEA

**Fig. 1:**
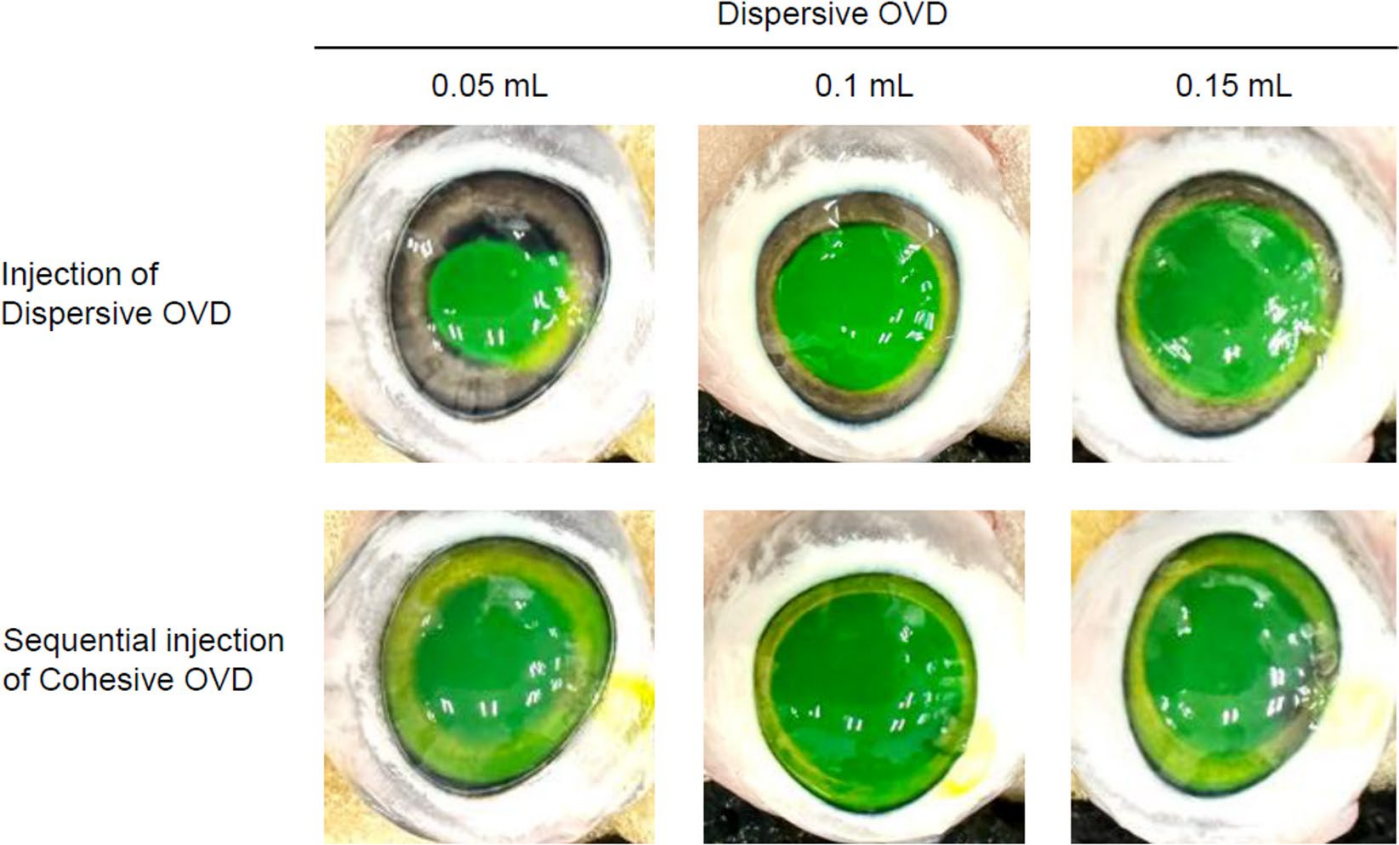
SST with different injection volumes of dispersive OVD. 0.05, 0.1, and 0.15 mL of stained dispersive OVD were injected into the anterior chamber (top). Subsequently, the cohesive OVD is injected under the dispersive OVD (bottom)

To qualitatively evaluate the retention of OVD in the eye, the dispersive OVD was stained with fluorescein (Fluores Ocular Examination Test Paper, Ayumi Pharmaceutical Corp., Tokyo, Japan). The fluorescein-containing portion of the strip was inserted into the tip of a syringe containing OVD, and stored for at least 2 days at room temperature. Stained dispersive OVD (0.05, 0.1, and 0.15 mL) was injected into the anterior chamber through a 2 mm or smaller side-port incision. At that time, images were taken by digital camera, and the area of each OVD was measured using ImageJ software to visually indicate the injected amount of the dispersed OVD (*n* = 5). Subsequently, the cohesive OVD was injected under the dispersive OVD until it leaked from the incision. Depending on the injection volume of the dispersive OVD, hereinafter, the groups are referred to as 0.05 mL-SST, 0.1 mL-SST, and 0.15 mL-SST respectively (Fig. 1). In addition, we also prepared porcine eyes in which the anterior chamber was filled with only 0.4 mL of the dispersive OVD, hereinafter referred to as 0.4 mL-Single. PEA was performed using a WHITESTAR SIGNATURE ™ PRO Phacoemulsification System (Johnson & Johnson, New Jersey, US). US (ultrasound) tip and sleeve used were 20-gauge Laminar^®^ Flow Phaco Tips 30° and 20-gauge Laminar^®^ sleeve (Johnson & Johnson), respectively. A 2.4 mm main corneal incision was created in the porcine eye, and OVD was aspirated for 0.5, 1, 2, 3, 4, 5 min with a US tip placed in the center of the anterior chamber without movement. As shown in Fig. S1, the OVD was removed to prevent perfusion from directly impinging on the corneal endothelium. PEA was performed using a peristaltic pump under the following conditions: (i) flow rate 35 mL/min, vacuum limit 250 mm Hg, bottle height 45 cm, or (ii) flow rate 45 mL/min, vacuum limit 500 mm Hg, bottle height 45 cm. During aspiration, the anterior chamber was irrigated with saline. To eliminate the possibility of additional factors influencing the outcome of this study, we did not perform US-assisted lens fragmentation, and therefore the US power was set to 0%. After aspiration, the eyeball with the cornea incised was immersed in 10 mL of distilled water overnight to elute the OVD. The OVD residual fraction for each PEA time was calculated, with the amount of CS in the OVD without PEA set at 100%.

### Measurement of chondroitin sulfate contained in dispersive OVD

Fluorescein-stained dispersive OVD was diluted with saline at ratios ranging from 100:1 to 1600:1. In this case, the theoretical CS content is 25–400 µg/mL. Each diluted solution was mixed with an equal volume of hydrochloric acid, and hydrolyzed at 110 °C for 2 h. Two hundred microliters of the mixture was passed through a C18 solid-phase extraction column and evaporated to dryness under reduced pressure and redissolved in distilled water. Because CS has a sulfate group in the molecule, the CS content was calculated by measuring the area under the curve of sulfate ions liberated from CS in the solution. CS content was estimated by using high performance liquid chromatography (HPLC) equipped with Shim-pack IC-SA3 (Shimadzu, Kyoto, Japan) under a constant flow rate at 0.8 mL/min of 3.6 mM sodium carbonate at 45 °C. Sulfate ion (FUJIFILM Wako Pure Chemical Corp., Osaka, Japan) was used as a standard solution. The CS content of the OVD solution after the PEA described above was also quantified using the same procedure. The method and principles of measuring sulfate ions using HPLC are illustrated in Fig. S2.

### Statistical analysis

At each time point, the residual fraction of OVD was compared among three volume groups, using one-way analysis of variance (ANOVA). When ANOVA was significant, pairwise group differences were examined using Tukey’s honestly significant difference (HSD) test to control the familywise error rate. The residual fraction and amount in the 0.1 mL-SST group performed under two aspiration conditions at each time point were compared using the Student’s t-test. We considered *p* values of less than 0.05 to be statistically significant.

## Results

**Fig. 2:**
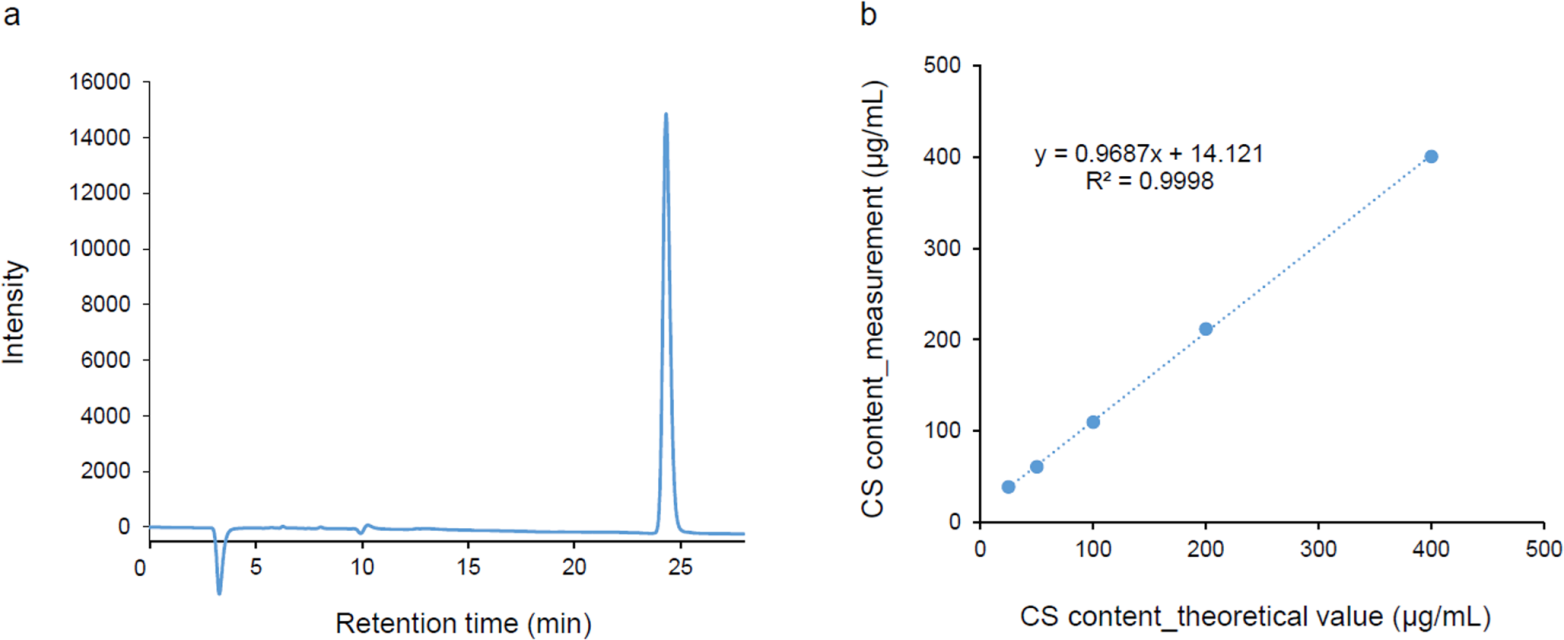
Determination of the amount of CS in dispersive OVD by measuring sulfate ions. (**a**) HPLC profile of sulfate ions. The peak at retention time 23.4 min comprised sulfate ions. (**b**) The vertical axis shows CS content calculated based on the sulfate ion peak area; the horizontal axis indicates the theoretical amount of CS contained in the dispersive OVD

As shown in Fig. 2a and b, the retention time of sulfate ions using column chromatography was 23.4 min. The CS content calculated based on the sulfate ion area under the curve was consistent with the theoretical value for the CS amount contained in the dispersive OVD (25–400 µg/mL) (R^2^ = 0.9998). It was also evident that fluorescein did not affect the determination of sulfate ions.

In the dispersive OVD with different injection volumes, the area of the circular OVD injected into the eye was 31.5 ± 5.8 mm^2^ for 0.05 mL of dispersive OVD, 74.7 ± 12.1 mm^2^ for 0.1 mL of dispersive OVD, and 91.2 ± 8.8 mm^2^ for 0.15 mL of dispersive OVD (*n* = 5), with diameters of approximately 6.3 mm, 9.7 mm, and 10.7 mm, respectively. Subsequently, injection of cohesive OVD resulted in the entire corneal endothelium being covered with dispersive OVD, regardless of the amount of dispersive OVD injected.

**Fig. 3:**
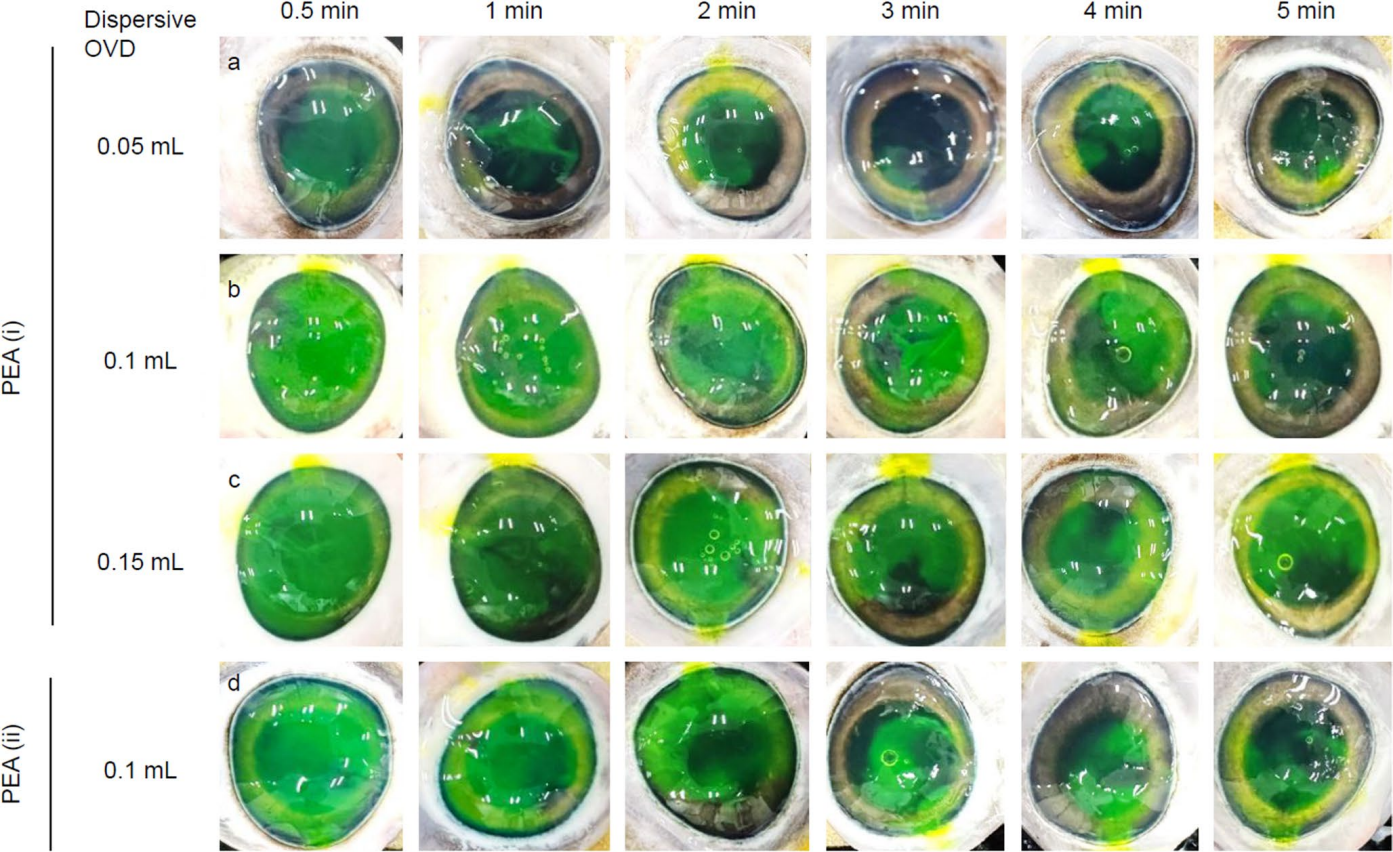
The ex vivo behavior of dispersive OVDs in the porcine anterior chamber. SST was performed with 0.05 (**a**), 0.1 (**b** or **d**), and 0.15 (**c**) mL of stained dispersive OVD. OVD was aspirated for 5 min through each US tip located at the center of the anterior chamber, without movement of the tip. For the PEA, flow rate, vacuum limit, and bottle height were set to the following two conditions: (i) 35 mL/min, 250 mm Hg, and 45 cm, or (ii) 45 mL/min, 500 mm Hg, and 45 cm

**Fig. 4:**
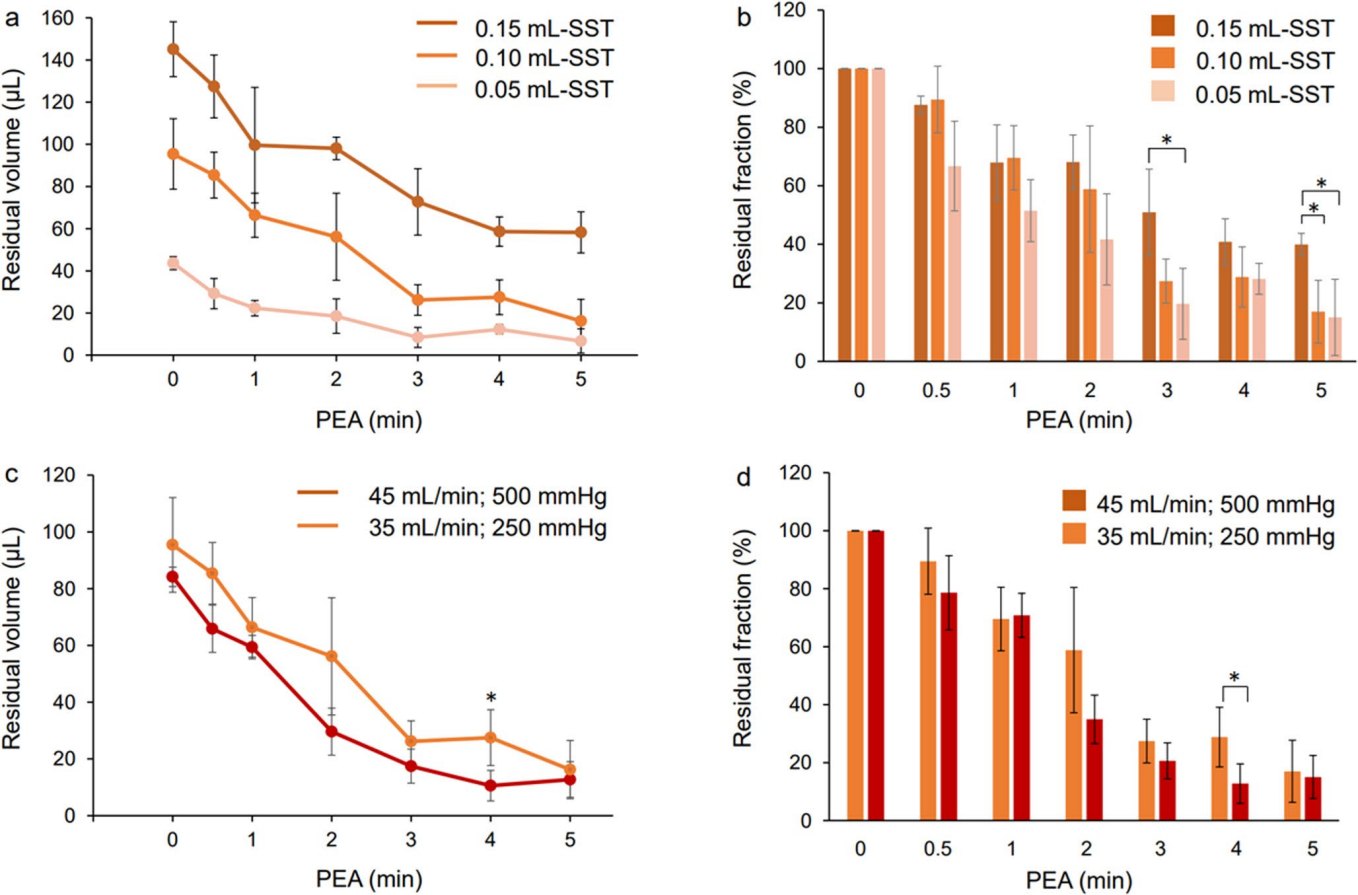
Residual volume and residual fraction of dispersive OVDs in the anterior chamber. (**a** and **b**) Retention durability of dispersive OVDs with different volumes injected into the anterior chamber. (**a**) Residual volume (µL) of dispersive OVD using SST at each PEA time point. (**b**) Residual fraction (%) of dispersive OVD using SST at each PEA time point. The OVD residual fraction for 0.05 mL SST was significantly lower than that for 0.15 mL-SST at 3 and 5 min of PEA (*p* < 0.05, Tukey’s HSD test). Values represent the mean ± standard deviation (*n* = 3 or 4). (**c** and **d**) Retention durability of dispersive OVD in the anterior chamber under different PEA conditions. PEA was performed under the following conditions: (i) flow rate 35 mL/min, vacuum limit 250 mm Hg, bottle height 45 cm, or (ii) flow rate 45 mL/min, vacuum limit 500 mm Hg, bottle height 45 cm. (**c**) Residual volume (µL) of 0.1mL dispersive OVD using SST at each PEA time point. (**d**) Residual fraction (%) of 0.1 mL dispersive OVD using SST at each PEA time point. There was no significant difference between PEA conditions (i) and (ii) except at 4 min of PEA (*p* < 0.05, t-test). Values represent the mean ± standard deviation (*n* = 3 or 4)

In the SST group, regardless of the amount of dispersive OVD injected, the OVD was removed in proportion to the PEA time, and the corneal endothelium uncovered by the OVD was partially exposed (Fig. 3). As shown in Figs. 3 and 4a, with 0.1 mL-SST, the corneal endothelium was generally covered until 2 min of PEA, and the amount of dispersive OVD remaining in the eye at that time was 56 µL or more. After 3 min of PEA, the remaining volume of OVD was less than 30 µL, revealing areas where the endothelium was not protected by the OVD. With 0.05 mL-SST, the remaining amount of dispersive-OVD was less than 30 µL after 0.5 min of PEA, and the corneal endothelium was almost unprotected after 1 min. With 0.15 mL-SST, 58 µL of dispersive-OVD remained after 5 min of PEA, and a sufficient amount remained in the eye to protect the corneal endothelium.

As shown in Fig. 4b, the OVD residual fraction with 0.05 mL-SST was significantly lower than with 0.15 mL-SST after 3 and 5 min of PEA (*p* < 0.05, Tukey’s HSD test). Furthermore, the residual fractions for 0.1 mL-SST and 0.15 mL-SST were comparable up to 4 min of PEA, but the fraction with the former was lower than with the latter at 5 min of PEA. (*p* < 0.05, Tukey’s HSD test). Using the SST, there was no significant difference in the residual fraction of OVD between injection volumes within 2 min of the start of PEA.

With 0.1 mL-SST, the residual amount and fraction of OVD was evaluated under two PEA conditions (Fig. 4c and d). The residual amount and fraction of OVD under both conditions was almost the same in both, except after 4 min of PEA (*p* < 0.05, t-test). Under PEA condition (ii), the pressure temporarily rose to 400 mmHg at the beginning of PEA, but thereafter it generally fluctuated between 100 and 200 mmHg and rarely rose significantly.

**Fig. 5:**
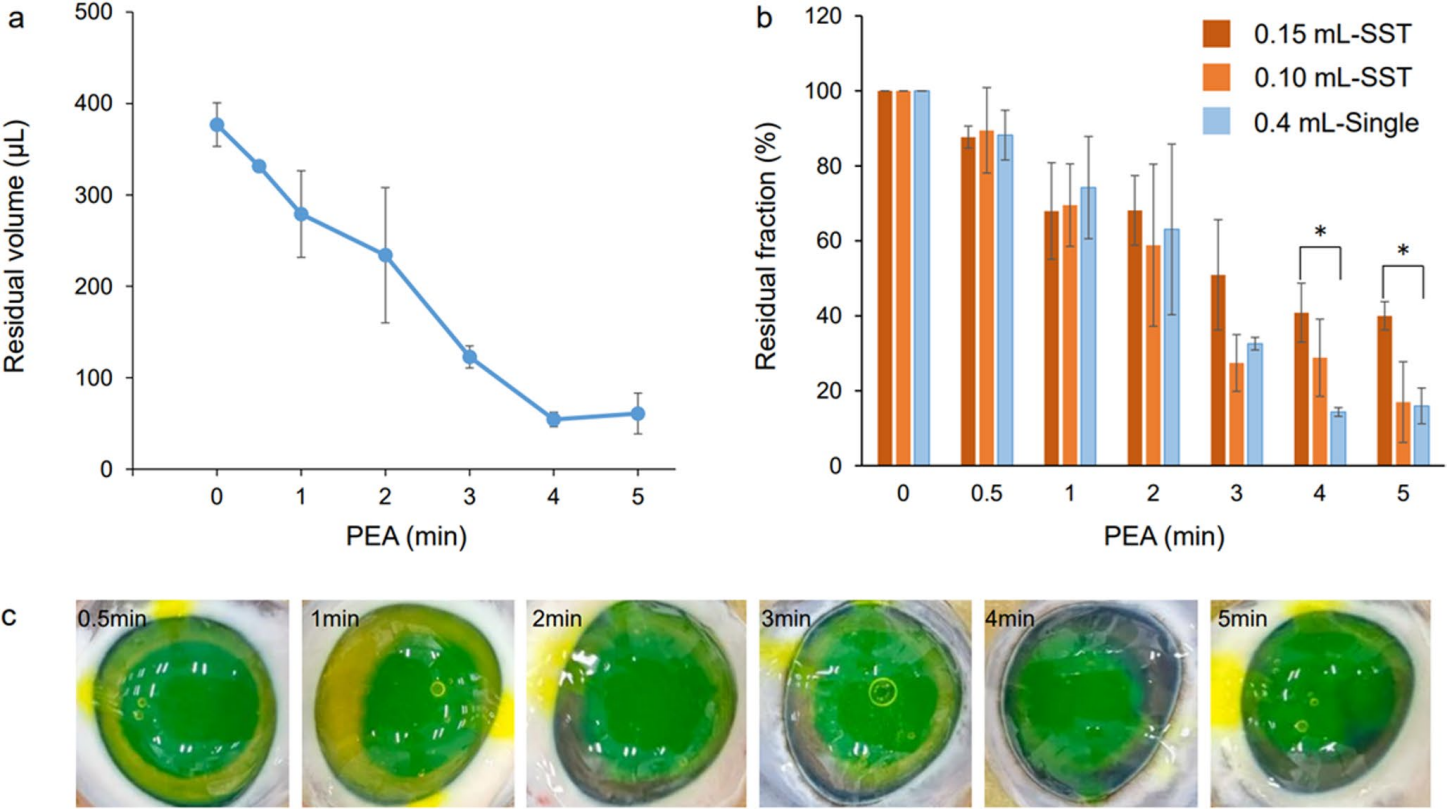
Retention durability of dispersive OVD alone in the anterior chamber. (**a**) Residual volume (µL) of 0.4 mL dispersive OVD alone at each PEA time point. (**b**) Comparison of residual fractions (%) between the SST group (0.15 mL-SST and 0.1 mL-SST) and 0.4 mL dispersive OVD alone at each PEA time point. Values for 0.15 mL-SST were higher than for the 0.4 mL-Single group at 4 and 5 min after PEA (*p* < 0.05, Tukey’s HSD test). (**c**) The behavior of 0.4 mL dispersive OVD alone in the porcine anterior chamber at each PEA time. PEA was performed under the following conditions: (i) flow rate 35 mL/min, vacuum limit 250 mm Hg, bottle height 45 cm. Values represent the mean ± standard deviation (*n* = 3 or 4)

In the 0.4mL-Single group, 60 µL of dispersive-OVD remained in the eye 5 min after PEA (Fig. 5a and c). In Fig. 5b, the OVD residual fraction for 0.15 mL-SST was higher than that for 0.4 mL-Single at 4 and 5 min after PEA (*p* < 0.05, Tukey’s HSD test). These results suggest that the use of a single agent significantly reduced OVD in the anterior chamber compared with the use of the SST. There was no significant difference in the OVD residual fraction between the 0.4 mL-Single and the 0.1 mL-SST groups, at any time.

## Discussion

In this study, we quantitatively demonstrated that the amount of OVD remaining in the eye after PEA differed depending on the injection volume of dispersive OVD, using the SST. In addition, by combining qualitative indicators, the remaining amount of OVD is visually displayed. The SST using a dispersive OVD of 0.1 mL or more protected the corneal endothelium for at least 2 min after PEA, regardless of flow rate or vacuum condition. In cases where PEA is prolonged, for example when the nucleus of the lens is hard, our evidence suggests that using 0.15 mL or more of dispersive OVD for the SST may be appropriate. In this study, 0.05 mL-SST did not sufficiently protect the corneal endothelium during PEA.

With the Shellgan^®^ used in this study, about 0.05 mL of agent was discharged for every 3 mm the piston traveled. The distance the piston moves is an indication of the amount of agent injected during surgery, but because the inner diameter of the syringe differs for each OVD product, this indication is limited to Shellgan^®^. Another indicator of the injection volume with OVD is the diameter of the OVD that expands into a circular shape after injection into the eye. If the diameter is 1 cm or more, it is expected that approximately 0.1 mL or more of OVD has been injected. These supportive data may serve as an indicator of the amount of OVD required for injection. However, it should be noted that the shape of the OVD injected into the eye during surgery may vary [12]. In several clinical studies evaluating the effects of SST on corneal endothelial protection, the injection volume of dispersive OVD was 0.1 mL, and that of cohesive OVD was 0.1 to 0.2 mL [13–15]. The porcine corneal area is approximately 136 mm^2^, while the human corneal area is approximately 100 mm^2^, as the porcine eye is slightly larger than the human eye [11, 16]. We believe that the amount of injected dispersive OVD required to protect the porcine corneal endothelium is also sufficient to protect the human eye.

Under two PEA conditions (i and ii), there was almost no difference in the amount of dispersive OVD remaining in the eye except at 4 min. Eda et al. reported that, using SST, a thin layer of dispersive OVD remains on the corneal endothelium under both low aspiration-low flow rate conditions (vacuum: 150 mmHg, bottle height: 80 cm, flow rate: 24 ml/min) and high aspiration-high flow rate conditions (vacuum: 300 mmHg, bottle height: 100 cm, flow rate: 30 ml/min) [17]. Under PEA condition (ii) of this study, vacuum pressure temporarily increased to 400 mmHg at the beginning of the PEA, which is thought to be due to the removal of cohesive OVD masses. It was suggested that setting the flow rate or vacuum condition may not affect OVD removal unless dispersive OVDs attached to the corneal endothelium are directly aspirated. A comparison of high-flow and low-flow phacoemulsification cataract surgery has shown similar anatomical outcomes, except for central macular thickness [18]. Conversely, it has been reported that a low perfusion pattern with low negative pressure is safe and effective for patients with corneal endothelial dysfunction complicated by cataracts [19]. Under either PEA condition, it is important to protect the corneal endothelium with an OVD.

Dispersive OVDs used alone provide enhanced corneal protection but require more time for removal. Excess intraocular OVD in the anterior chamber prolongs the surgical time and manipulation for removal. Conversely, SST promotes deepening and stabilization of the anterior chamber, offering particular advantages during delicate maneuvers such as capsulorhexis. Simultaneously, the corneal protective effect provided by the dispersive OVD is maintained [9].

Suzuki et al. proposed that the SST better coats the corneal endothelium, compared with dispersive OVD alone. This is because the cohesive OVD can push the dispersive OVD consistently against the corneal endothelium, with the use of the SST [20]. The choice between single agents and the SST is expected to depend on the case, surgical conditions, and the surgeon’s technique. Alternatively, in some cases, an additional OVD may be injected after SST. According to Lin et al., the lens nucleus was removed by PEA using the SST, and subsequently dispersive OVD was injected into the anterior chamber again to support the capsular bag [19]. In addition, if PEA takes a long time due to the presence of a severe cataract, it may be possible to inject dispersive OVD alone after SST. If additional amounts of dispersive OVD are injected, the time required for OVD removal must also be considered.

Several previous studies have reported the time required for removal of 3% HA / 4% CS combinations or PEA time (Table 1). Although the OVD removal time or PEA time is approximately 1 to 2 min, most studies evaluating the intraocular residual volume of OVDs have been qualitative. In clinical trials, it is expected that visual assessment of the time required for removal of colorless and transparent OVDs will be difficult. Furthermore, because the PEA conditions, equipment, and OVD injection volume varied, it is difficult to compare OVD removal times across studies. In this study, the injection volume was made uniform and the remaining volume was quantitatively evaluated, making it possible to compare the remaining volume of OVD over PEA time.

**Table 1:** Removal time of 3% HA / 4% CS combination or PEA time and test conditions

Author	Human/ Animal	SST /Single	Removal or PEA times (sec)	US power (%)	Vacuum (mmHg)	Flow rate (mL/min)	Bottle height (cm)	Ref.
Miller et al. 1999	Human	Single	75	-	-	-	-	21]
Ishikawa et al., 2001	Human	SST	93	-	-	-	-	[22]
Vajpayee et al., 2005	Human	Single	66	-	-	-	-	[23]
Nakagawa et al., 2022	Human	Single	80 or 98	40/50	50/350	21/35	-	[24]
Oshika et al.,2006	Porcine	Single	100–150	30	300	35	85	[25]
Miyajima et al., 2006	Porcine	Single	53	40	380	20/40	78	[26]
Daf et al., 2024	Rabbit	SST/Single	SST:71 Single: 81	-	500	35	-	[27]

This is the first study to quantify the intraocular residual amount of 3% HA / 4% CS combinations by measuring the sulfur content of CS. While the study provides valuable insights, it has some limitations. In this study, the US tip was not moved to eliminate subjective evaluation, but in actual clinical surgery, it is moved within the eye. Furthermore, no lens fragmentation was performed. In the future, we would like to evaluate the amount of OVD remaining in the eye according to the surgical procedure and operation. In this study, we established a method of accurately quantifying the amount of OVD remaining in the eye, but we were unable to evaluate the amount of OVD coated on the corneal endothelium. In Figs. 4 and 5, more than 80% of the data at each time point for each dose have a standard deviation of less than 15, indicating high homogeneity. However, since the sample size for this study was *n* = 3–4, a larger sample size is often better. Further research is needed to optimize the use and selection of OVDs in cataract surgery.

The findings of this study may be clinically relevant. Using the soft shell technique with 0.1 mL of dispersive OVD is expected to be sufficient for standard cataract surgery. If PEA takes more than 2 min, injecting more than 0.1 mL of dispersive OVD should be considered. The relationship between the injected amount of dispersive OVD and PEA time obtained in this study can be useful in understanding and predicting the behavior of OVD in the eye during surgery. Evidence-based information may assist surgeons in making a sound medical decision.

With the increasing number of OVD options available for cataract surgery, it is essential to gain a comprehensive understanding of the characteristics of each OVD, the time required for its removal and the residual amount of OVD remaining in the eye. A clear understanding of the appropriate injection volume and residual intraocular amount plays a critical role in protecting the corneal endothelium and ensuring the overall safety and success of the surgery.

## Supplementary Information

Below is the link to the electronic supplementary material.

Supplementary Material 1

## Data Availability

No datasets were generated or analysed during the current study.
