# Supplementary material for: Quantitative assessment of ophthalmic viscosurgical device retention during phacoemulsification and aspiration: an ex vivo analysis
Source: Graefes Arch Clin Exp Ophthalmol. 2025 Dec 18;264(4):1167–75. doi: 10.1007/s00417-025-07073-4 (PMC13002746; doi:10.1007/s00417-025-07073-4)
Supplement: Supplementary file 1 — Supplementary Material 1 [file 417_2025_7073_MOESM1_ESM.docx]

**Supplementary material**


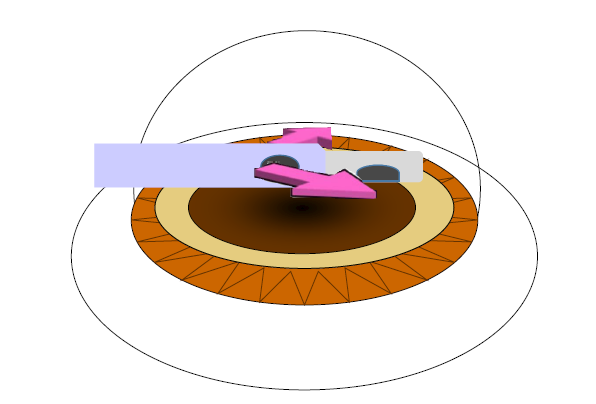


**Figure S1. Schematic illustration of perfusion in the eye.** The direction of perfusion is indicated by the pink arrow. The perfusion was directed laterally to prevent it impinging directly on the corneal endothelium.

**
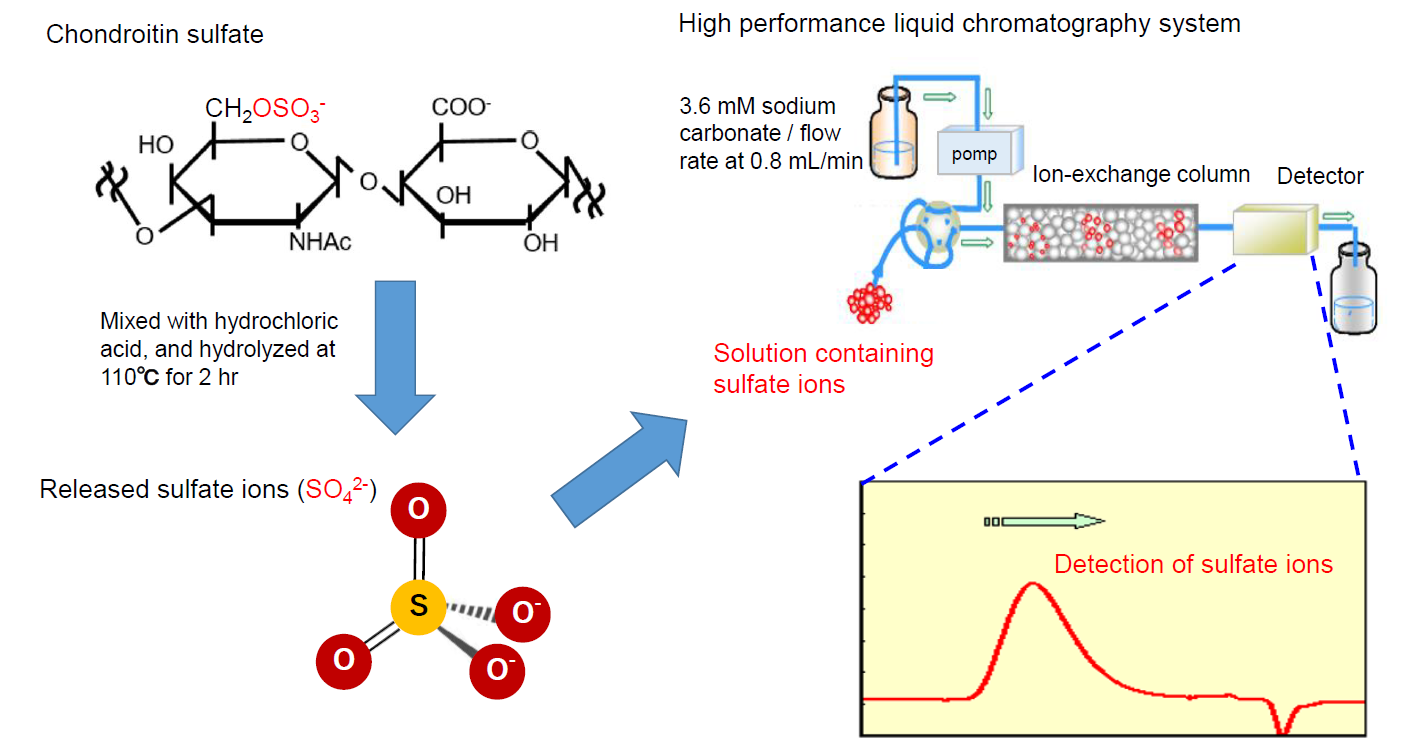
**

**Figure S2**. **Schematic illustration of** **the method and principles of measuring sulfate ions using high performance liquid chromatography.** First, sulfate ions were liberated from chondroitin sulfate. Next, the solution containing sulfate ions was injected into a high performance liquid chromatography system equipped with an ion-exchange column using 3.6 mM sodium carbonate at a constant flow rate of 0.8 mL/min. The chondroitin sulfate content was calculated based on the sulfate ion area under the curve.
